# Supplementary material for: Mammalian forelimb evolution is driven by uneven proximal-to-distal morphological diversity
Source: eLife. 2023 Jan 26;12:e81492. doi: 10.7554/eLife.81492 (PMC9908075; doi:10.7554/eLife.81492)
Supplement: Supplementary file 2. — (a) Linear measurements obtained for each specimen extracted either by landmark positioning in 3D digital models or by direct caliper measurements (illustrated in Figure 2). (b) Fits of linear models of evolution for each bone, highlighting in bold the best model fitted according to generalized information criterion (GIC) and loglikelihood (logLik). σ²=mean evolutionary rate, α=attraction toward optimum, stat.var.=mean stationary variance. (c) Empirical values from PGLS regression computing for body mass in the geometric means. (d) Empirical values from PGLS regression without body mass in the geometric means. [file elife-81492-supp2.docx]

**Supplementary file 2**

**Supplementary file 2a.** Linear measurements obtained for each specimen extracted either by landmark positioning in 3D digital models or by direct caliper measurements (illustrated in Figure 2).

**I) Humerus length**

Landmark 1 - Midpoint of the intertubercular groove

Landmark 2 - Distal end of the trochlea

**II) Humerus proximal width**

Landmark 3 - Right edge just above the neck of the humerus

Landmark 4 - Left edge just above the neck of the humerus

**III) Humerus mid shaft width**

Landmark 5 - Right edge of central axis in anterior view

Landmark 6 - Left edge of central axis in anterior view

**IV) Humerus distal width**

Landmark 7 - Right edge of lateral epicondyle

Landmark 8 - Left edge of medial epicondyle

**V) Humerus height**

Landmark 9 - Right edge of central axis in lateral view

Landmark 10 - Left edge of central axis in lateral view

**VI) Radius length**

Landmark 11 – Sulcus in the radius head

Landmark 12 – Distal end of styloid process

**VII) Radius proximal width**

Landmark 13 – Rightest edge under epiphysis suture of radius head

Landmark 14 – Leftist edge under epiphysis suture of radius head

**VIII)** **Radius mid shaft width**

Landmark 15 - Right edge of central axis in anterior view

Landmark 16 - Left edge of central axis in anterior view

**IX)** **Radius distal width**

Landmark 17 – Rightest edge above epiphysis suture of distal radius

Landmark 18 – Leftist edge above epiphysis suture of distal radius

**X) Radius height**

Landmark 9 - Right edge of central axis in lateral view

Landmark 10 - Left edge of central axis in lateral view

**XI)** **Third metacarpal length**

Landmark 21 – Sulcus of articulation with magnum carpal

Landmark 22 – Proximal-most tip of epiphysis

**XII)** **Third metacarpal proximal width**

Landmark 23 – Rightest edge under the suture of the proximal epiphysis

Landmark 24 – Leftist edge under the suture of the proximal epiphysis

**XIII) Third metacarpal mid shaft width**

Landmark 25 - Right edge of central axis in dorsal view

Landmark 26 - Left edge of central axis in dorsal view

**XIV)** **Third metacarpal distal width**

Landmark 27 – Rightest edge above distal epiphysis suture

Landmark 28 – Leftist edge above distal epiphysis suture

**XV) Third metacarpal height**

Landmark 29 - Right edge of central axis in lateral view

Landmark 30 - Left edge of central axis in lateral view

**XVI)** **Digit III phalanx I length**

Landmark 31 – Sulcus of articulation with third metacarpal

Landmark 32 – Sulcus of articulation with second phalanx

**XVII) Digit III phalanx proximal width**

Landmark 23 – Rightest edge under the suture of the distal epiphysis

Landmark 24 – Leftist edge under the suture of the distal epiphysis

**XVIII) Digit III phalanx mid shaft width**

Landmark 29 - Right edge of central axis in dorsal view

Landmark 30 - Left edge of central axis in dorsal view

**XIX)** **Digit III phalanx distal width**

Landmark 37 – Rightest edge above distal epiphysis suture

Landmark 38 – Leftist edge above distal epiphysis suture

**XX)** **Digit III phalanx height**

Landmark 39 - Right edge of central axis in lateral view

Landmark 40 - Left edge of central axis in lateral view

**Supplementary file 2b.** Fits of linear models of evolution for each bone, highlighting in bold the best model fitted according to generalized information criterion (GIC) and loglikelihood (logLik). σ² = mean evolutionary rate, α = attraction toward optimum, stat.var. = mean stationary variance.

|  | **Best-fit model rank** | **GIC** | **lnL** | **σ²** | **α** | **stat. var.** |
| --- | --- | --- | --- | --- | --- | --- |
| **Humerus** | **OU** | **-3494.708** | **1796.783** | **1.59E-03** | **0.020** | **0.039** |
|  | BM | -3158.830 | 1647.660 | 1.13E-03 | - | - |
|  | EB | -3156.829 | 1647.660 | 1.13E-03 | - | - |
| **Radius** | **OU** | **-3244.176** | **1660.549** | **1.83E-03** | **0.022** | **0.041** |
|  | BM | -2374.789 | 1239.853 | 1.28E-03 | - | - |
|  | EB | -2845.109 | 1468.898 | 1.28E-03 | - | - |
| **Metacarpal** | **OU** | **-2645.917** | **1382.228** | **2.02E-03** | **0.017** | **0.060** |
|  | BM | -2374.789 | 1239.853 | 1.49E-03 | - | - |
|  | EB | -2372.786 | 1239.851 | 1.49E-03 | - | - |
| **Phalanx** | **OU** | **-2767.643** | **1444.173** | **2.70E-03** | **0.024** | **0.055** |
|  | BM | -2333.077 | 1227.580 | 1.64E-03 | - | - |
|  | EB | -2331.071 | 1227.576 | 1.64E-03 | - | - |

**Supplementary file 2c.** Empirical values from PGLS regression computing for body mass in the geometric means.

**Supplementary file 2d.** Empirical values from PGLS regression without body mass in the geometric means.
